# Pretransplant HbA1c Is a Useful Predictor for the Development of New-Onset Diabetes in Renal Transplant Recipients Receiving No or Low-Dose Erythropoietin

**DOI:** 10.1155/2014/436725

**Published:** 2014-10-16

**Authors:** Kazuaki Tokodai, Noritoshi Amada, Izumi Haga, Atsushi Nakamura, Toshiaki Kashiwadate, Naoki Kawagishi, Noriaki Ohuchi

**Affiliations:** ^1^Department of Surgery, Sendai Shakaihoken Hospital, Sendai 980-8574, Japan; ^2^Division of Transplantation, Reconstruction and Endoscopic Surgery, Tohoku University Hospital, 1-1 Seiryo-machi, Aoba-ku, Sendai 980-8574, Japan

## Abstract

*Aims*. To evaluate the predictive power of pretransplant HbA1c for new-onset diabetes after transplantation (NODAT) in kidney transplant candidates, who had several predispositions for fluctuated HbA1c levels. *Methods*. We performed a retrospective study of 119 patients without diabetes who received kidney transplantation between March 2000 and January 2012. Univariate and multivariate logistic regression analyses were used to investigate the association of several parameters with NODAT. Predictive discrimination of HbA1c was assessed using a receiver-operating characteristic curve. *Results*. Seventeen patients (14.3%) developed NODAT within 1 year of transplantation. Univariate logistic regression analysis revealed that recipient age, gender, and HbA1c were predictors of NODAT. In the multivariate analysis, the association between pretransplant HbA1c and NODAT development did not reach statistical significance (*P* = 0.07). To avoid the strong influence of high-dose erythropoietin on HbA1c levels, we performed subgroup analyses on 85 patients receiving no or low-dose (≤6000 IU/week) erythropoietin. HbA1c was again an independent predictor for NODAT. Receiver-operating characteristic analysis revealed a cut-off value of 5.2% with an optimal sensitivity of 64% and specificity of 78% for predicting NODAT. *Conclusions*. Our results reveal that the pretransplant HbA1c level is a useful predictor for NODAT in patients receiving no or low-dose erythropoietin.

## 1. Introduction

A serious and frequent complication of transplantation—new-onset diabetes after transplantation (NODAT)—is associated with increased cardiovascular morbidity and mortality that is observed in kidney transplant patients [1, 2] as well as with decreased graft and patient survival [3–5]. Thus, pretransplant identification of patients at high risk of developing NODAT would be greatly advantageous by enabling the modification of NODAT by the use of less diabetogenic immunosuppressive drugs or implementation of lifestyle-change interventions. Several clinical trials have revealed that lifestyle-change interventions were effective in preventing type 2 diabetes mellitus [6, 7] and that they may be applicable in NODAT cases too. We previously reported that the posttransplant increase in body mass index and body fat percentage is associated with the development of NODAT [8, 9], indicating that lifestyle-change interventions are useful for preventing NODAT.

The postload plasma glucose level during an oral glucose tolerance test (OGTT) is a strong predictor for the development of diabetes in the future [10, 11]. Furthermore, we have recently reported that OGTT can be a useful predictor of NODAT development [12]. However, OGTT is time consuming and forces patients to fast overnight. HbA1c is much easier to test and examine than OGTT and has been increasingly used for assessing chronic glycemia in patients with diabetes, diagnosing diabetes mellitus, and identifying those who may be at high risk for developing diabetes in the future [13–16]. Furthermore, several studies showed the efficacy of HbA1c in the detection of posttransplant diabetes mellitus [17–19]. However, HbA1c values are strongly influenced by anemia and erythropoietin (EPO) treatment [20–22]. Further, kidney transplant candidates are frequently administered EPO injections for the treatment of anemia; hence, the pretransplant HbA1c level should be cautiously interpreted. Although Tatar et al. showed that the pretransplant HbA1c level can be used to identify the high-risk group of NODAT [23], the utility of HbA1c as a predictor for the development of NODAT has not been completely elucidated.

In our previous study, all patients who developed NODAT had received tacrolimus-based immunosuppressive treatment [8]. Hence, in this study, we evaluated the predictive power of the pretransplant HbA1c level for the development of NODAT in patients receiving tacrolimus-based immunosuppressive treatment.

## 2. Patients and Methods

### 2.1. Patient Selection

**Figure 1:**
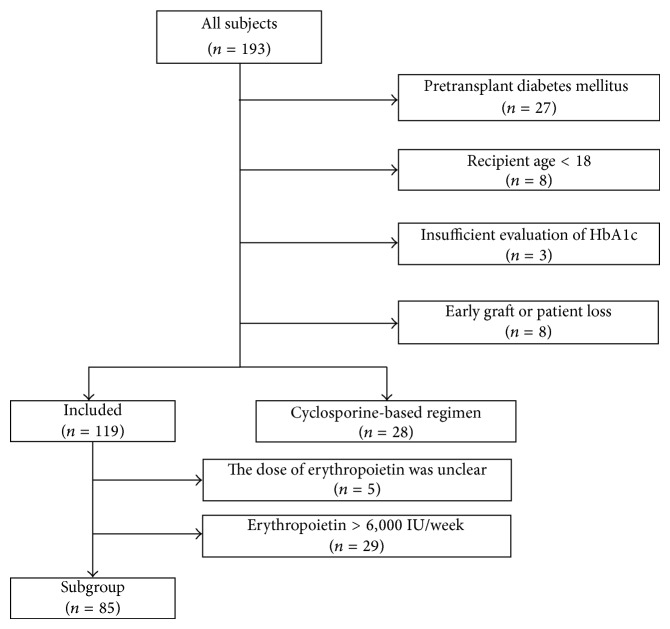
Flowchart of patient enrollment.

We retrospectively identified 193 consecutive patients who received living donor kidney transplants in our hospital between March 2000 and January 2012. Seventy-four patients were excluded from this study for the following reasons: pretransplant diabetes mellitus (*n* = 27), recipient age < 18 years (*n* = 8), insufficient data on HbA1c (*n* = 3), early graft or patient loss (*n* = 8), and cyclosporine-based immunosuppressive regimen (*n* = 28). Pretransplant diabetes was defined as the use of insulin or oral antihyperglycemic medications or fasting plasma glucose levels ≥ 126 mg/dL. Finally, a total of 119 patients were included in this study (Figure 1). For subgroup analysis, we identified 85 patients receiving no or low-dose (≤6,000 IU/week) EPO. To avoid detection bias, systematic, standardized, and periodic examinations of posttransplant glucose tolerance were performed on all patients regardless of pretransplant HbA1c levels. HbA1c was measured basically within 3 months prior to transplantation by high performance liquid chromatography (HPLC) method and estimated as a National Glycohemoglobin Standardization Program (NGSP) value. The HbA1c (Japan Diabetes Society (JDS)) was converted to HbA1c (NGSP) using the officially certified equation: NGSP (%) = 1.02 × JDS (%) + 0.25% [24]. The clinical data were collected retrospectively from the hospital data system and from clinical records in August 2013. The local institutional internal review board approved this study.

### 2.2. Immunosuppressive Regimens

Patients received immunosuppressive treatment consisting of prednisolone, tacrolimus, mycophenolate mofetil or mizoribine, and basiliximab. Prednisolone was started at 1 mg/kg with subsequent tapering to 0.2 mg/kg 1 month after transplantation. Tacrolimus was initiated 2 days before transplantation and adjusted to maintain the initial trough level of 10–12 ng/mL and the long-term target trough level of 6–8 ng/mL. Mycophenolate mofetil or mizoribine was started on the day after transplantation. Basiliximab 20 mg was administered intravenously on days 0 and 4. Acute rejection was confirmed by graft biopsy and treated with intravenous methylprednisolone (250 or 125 mg/day) for 3 days followed by deoxyspergualin 5 mg/[kg*·*day] for 5 days.

### 2.3. Definition of NODAT

NODAT was defined according to the American Diabetes Association [25] as the presence of diabetes symptoms in addition to casual plasma glucose levels ≥ 200 mg/dL or fasting plasma glucose levels ≥ 126 mg/dL. Fasting was defined as the absence of caloric intake for at least 8 h. In this study, patients with transient elevations of fasting plasma glucose levels immediately after transplantation or during times of acute illness were not diagnosed with NODAT. Patients who presented with impaired fasting glucose were introduced to diabetologists for further examinations. Patients who started to receive insulin or oral antihyperglycemic medications after transplantation were also diagnosed as NODAT.

### 2.4. EPO Injection

All doses of EPO were presented as recombinant human EPO per week. For the conversion of darbepoetin alfa dosage to recombinant human EPO, a ratio of 1 *μ*g darbepoetin alfa to 200 IU recombinant human EPO ratio was used [26]. Recombinant EPO is generally used with a dose of 4,500, 6,000, or 9,000 IU/week. In this study, low-dose and high-dose EPO were defined as 0 < EPO ≤ 6,000 IU/week and EPO > 6,000 IU/week, respectively.

### 2.5. Statistical Analysis

Data are expressed as means (±standard deviation) or median (range) as appropriate. Statistical significance was determined using Student's *t*-test for normally distributed data, Wilcoxon's signed rank test for skewed data, and Fisher's exact test for dichotomous data. Univariate and multivariate logistic regression analyses were used to assess the association of several parameters with the incidence of NODAT. Predictive discrimination of HbA1c was assessed using a receiver-operating characteristic (ROC) curve on the basis of maximizing sensitivity and specificity. Analyses were performed using JMP Pro 10 (SAS Institute, Cary, NC). A value of *P* < 0.05 was considered significant.

## 3. Results

### 3.1. Patient Demographics

The baseline data of the patients who did and did not develop NODAT are presented in Table 1. Of the 119 patients included in this study, 17 patients (14.3%) developed NODAT within 1 year of transplantation. Patients who developed NODAT were significantly older than those who did not develop NODAT. There were no differences in donor age, gender, cause of chronic renal failure, or pretransplant body mass index.

### 3.2. Predictors of NODAT

Univariate logistic regression analysis showed that recipient age, gender, and HbA1c were significant predictors for the development of NODAT. In the multivariate analysis, only the recipient age was an independent predictor of NODAT. The association between the pretransplant HbA1c level and development of NODAT did not reach statistical significance (*P* = 0.07), after adjusting for recipient age and gender, the 2 factors of *P* < 0.05 (Table 2).

### 3.3. Subgroup Analyses for the Patients Receiving No or Low-Dose EPO

**Figure 2:**
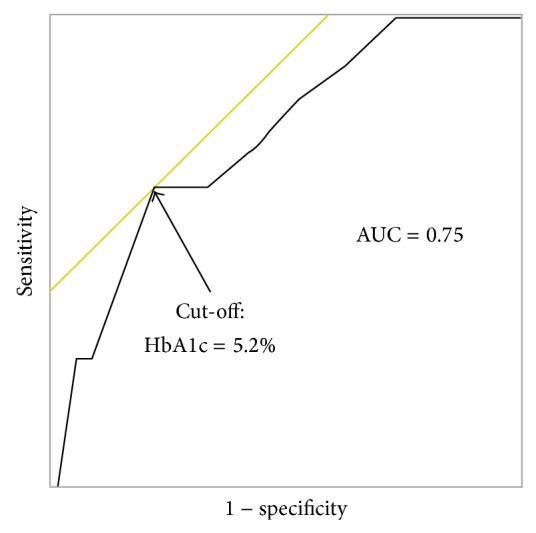
The receiver-operating characteristic curve of pretransplant HbA1c. The cut-off point of the HbA1c level was 5.2%, which was derived on the basis of maximizing sensitivity and specificity. AUC: area under the receiver-operating characteristic curve.

For 29 of the 119 patients included in this study, EPO doses were > 6,000 IU/week; the EPO doses of 5 patients were not available. Therefore, these 34 patients were excluded from the subgroup analyses of patients receiving no or low-dose EPO. Of the 85 patients that were included, 11 patients (12.9%) developed NODAT. Multivariate logistic regression subanalysis revealed that the pretransplant HbA1c level was an independent predictor for the development of NODAT (Table 3). The ROC analyses showed that the area under the ROC curve was 0.75 and the cut-off level of HbA1c which gave the maximum sensitivity and specificity was 5.2% (Figure 2). The sensitivity, specificity, positive predictive value, and negative predictive value of HbA1c at the cut-off point were 64%, 78%, 30%, and 94%, respectively (Table 4).

## 4. Discussion

In this study, we found that even in patients with chronic renal failure, who had several predispositions for fluctuated HbA1c levels, pretransplant HbA1c was associated with NODAT development; moreover, pretransplant HbA1c levels were an independent predictor of NODAT development in patients receiving no or low-dose EPO. Our subgroup analysis further revealed that the cut-off value for HbA1c was 5.2% and indicated that the risk of NODAT development was significantly higher in patients with HbA1c levels above this cut-off.

Pretransplant HbA1c levels were a significant predictor of NODAT for patients receiving no or low-dose EPO; however, this association was not observed during the whole group analysis. This inconsistency might be due to a high degree of variability in HbA1c levels in patients receiving high-dose EPO. Uzu et al. reported that high-dose EPO strongly influenced HbA1c levels [27], and therefore pretransplant HbA1c levels might be difficult to apply to these patients.

Our study has several points in common with those of a study by Tatar et al. that revealed the predictive value of pretransplant HbA1c for NODAT development within 1 year after kidney transplantation. Both the studies excluded recipients aged < 18 years and included recipients with similar baseline characteristics, including equivalent age, sex, and pretransplant HbA1c level. The major difference between these two studies was the study design: our study excluded patients who had received a cyclosporine-based immunosuppressive regimen because our previous study had showed that all patients who developed NODAT had received a tacrolimus-based immunosuppressive regimen [12]. Furthermore, in our study, the incidence rate of NODAT development was lower (14.3%) than that in Tatar's study (25.9%), and pretransplant BMI and fasting plasma glucose levels were not associated with NODAT development. These inconsistencies might be due to the difference in race, considering that all the included patients in our study were Japanese with low BMIs. A weakness of our study is that the predictive value of pretransplant HbA1c was determined only for the subgroup patients who did not receive EPO or patients who received only low-dose EPO. However, we believe that this point is also an original strong point of our study because most of the kidney transplant candidates received EPO therapies, and pretransplant HbA1c level should be interpreted after considering the EPO dose.

The cut-off point of 5.2%, which was derived by the subgroup analysis on the basis of maximizing sensitivity and specificity, was considered clinically acceptable, because it could identify the low- and high-risk groups with reasonable predictive values as a predictor of short-term NODAT development. The risk of NODAT development in patients receiving no or low-dose EPO with an HbA1c ≥ 5.2% was 6.34-fold higher than in patients with an HbA1c < 5.2%, which is higher than the reported odds ratio of impaired glucose tolerance based on a OGTT [28]. According to the American Diabetes Association, prediabetes is defined as 5.7% ≤ HbA1c ≤ 6.4% in the general population [13]. The cut-off value derived from this study was lower than that for the general population, which is most likely due to the influence of EPO; this is consistent with reports suggesting that the target HbA1c level should be lowered to 5.1% for patients with hematocrit < 30 and EPO ≥ 100 IU/[kg*·*week] [27]. The AUC of 0.75, sensitivity of 64%, and specificity of 78% compare favorably with previously reported values in the general population [15], indicating that HbA1c values are indeed useful and can be applied to clinical practices in transplant settings, although the cut-off level of 5.2% needs to be further evaluated.

Although several reports suggest that the HbA1c level can be interpreted after using a correction formula (HbA1c ∗ 1.19 in those with low EPO dosages, i.e., <100 IU/[kg*·*week], and HbA1c ∗ 1.38 in those with high EPO dosages, i.e., ≥100 IU/[kg*·*week]) [27], this formula is complicated to use in the clinical setting, as it requires clinicians to calculate the corrected HbA1c value each time. This formula is even less practical with regard to recently developed EPO drugs that have a longer elimination half-life because the dosage for these new drugs must first be converted into the comparable recombinant human EPO dose before the correction formula can be applied. In this study, 29 out of a total of 119 patients received high-dose EPO (>6,000 IU/week) therapy. By excluding this minority group, pretransplant HbA1c was an independent predictor for NODAT in the subgroup multivariate analysis. These results indicate that the pretransplant HbA1c level, which has been considered difficult to interpret in patients with chronic renal failure, can be used as a useful predictor of NODAT by excluding patients receiving maximum dose EPO.

The uremic state was also a well-known factor to affect the HbA1c levels measured by HPLC method. Carbamylated hemoglobin, which was greatly elevated in uremia, was significantly correlated with chromatographically determined glycosylated hemoglobin [29]. Indeed, HbA1c levels measured by HPLC should be interpreted with much caution in uremia. On the other hand, Smith et al. also reported that hemodialysis had no effect on the HbA1c levels measured by HPLC although the slight increases were observed in the settings of chronic renal failure and continuous ambulatory peritoneal dialysis [29]. Furthermore, HbA1c values measured by HPLC have been widely utilized for patients with chronic renal failure [22, 27] and shown to be associated with increased death risk in the patients undergoing maintenance hemodialysis [30, 31]. Based on these results, we believe that HbA1c can be a useful tool even for patients with uremia.

In this study, fasting plasma glucose levels were comparable between the groups, whereas in the study by Tatar et al., pretransplant fasting blood glucose levels were reported as an independent predictor of NODAT development [23]. As the predictive value of fasting plasma glucose for the development of type 2 diabetes remains controversial [32, 33], its predictive value for NODAT development needs to be further investigated. Hepatitis C virus (HCV) infection is also an important risk factor of the development of type 2 diabetes, as we have previously reported [12]. In this study, however, only 3 of the 119 patients had HCV infections; hence, the association between HCV infections and NODAT development could not be statistically investigated.

Our study has several limitations. This was a retrospective single-center study, and the sample size was small. Because of the small sample size, the predictive power of HbA1c according to the EPO dose could not be fully compared. For the same reason, we could not fully assess the utility of HbA1c in patients receiving high-dose EPO. Furthermore, we could not compare the efficacy of HbA1c with that of other indexes such as oral glucose tolerance tests. However, we believe that pretransplant HbA1c levels are easy to obtain and can be a useful tool to identify patients at high risk for the development of NODAT.

Our results reveal that the pretransplant HbA1c level is an important predictor for the development of NODAT for patients receiving EPO doses of ≤6,000 IU/week. There are several restrictions for the use of the HbA1c level as a predictor of NODAT before kidney transplantation, especially in patients receiving high-dose EPO treatment. In the future, longer-term prospective studies with larger sample sizes should be conducted to confirm our findings and examine the use of the pretransplant HbA1c level for patients receiving high-dose EPO. The clinical impact of improving the identification of patients at high risk of developing NODAT prior to transplantation is high, as it would allow for the individual adaptation of immunosuppressive treatments and implementation of lifestyle-change interventions.

## Figures and Tables

**Table 1:** Comparison of patients who developed NODAT^a^ (NODAT+) to those who did not (NODAT−).

Variable	NODAT+ (*n* = 17)	NODAT− (*n* = 102)	*P* value
Recipient age [y]	47.5 (10.5)	38.7 (11.7)	<0.005
Recipient gender (M/F)	(15/2)	(64/38)	0.05
Donor age [y]	53.4 (7.9)	55.6 (10.2)	0.38
Donor gender (M/F)	(4/13)	(36/66)	0.42
Fasting plasma glucose [mg/dL]	82.9 (6.9)	80.1 (9.0)	0.22
HbA1c [%]	5.2 (0.45)	4.9 (0.45)	<0.05
Hemoglobin [g/L]	10.6 (2.4)	10.4 (1.8)	0.60
EPO^b^ (none/low-dose^c^/high-dose^d^)	(1/11/4)	(15/59/25)	0.74
Cause of chronic renal failure			0.50
Glomerulonephritis	13 (76%)	62 (61%)	
Polycystic kidney disease	0 (0%)	4 (4%)	
Hypertension/nephrosclerosis	1 (6%)	4 (4%)	
Other/unknown	3 (18%)	32 (31%)	
Pretransplant BMI^e^	21.1 (2.6)	21.2 (3.1)	0.95
Dialysis period [mo]	24 (0–171)	15 (0–198)	0.23
Hepatitis C positive	2 (12%)	1 (1%)	0.05
ABO incompatible	3 (18%)	15 (15%)	0.72

^a^New-onset diabetes after transplantation.

^b^Erythropoietin.

^c^0 < EPO ≤ 6,000 IU/week.

^d^EPO > 6,000 IU/week.

^e^Body mass index [kg/m^2^].

**Table 2:** Risk factors for NODAT^a^: univariate and multivariate analyses.

Variable	Univariate analysis			Multivariate analysis		
	OR^b^	95% CI^c^	*P* value	OR	95% CI	*P* value
Recipient age [y]	1.05	1.02–1.12	<0.005	1.06	1.01–1.12	<0.05
Recipient gender	4.45	1.17–29.2	<0.05	4.10	0.99–28.5	0.05
Donor age [y]	0.98	0.93–1.03	0.39			
Pretransplant BMI^d^	0.98	0.83–1.17	0.95			
FPG^e^ [mg/dL]	1.04	0.98–1.10	0.23			
HbA1c [%]	3.99	1.34–12.8	<0.05	3.09	0.90–11.1	0.07
Dialysis period [mo]	1.01	0.99–1.02	0.26			

^a^New-onset diabetes after transplantation.

^b^Odds ratio.

^c^95% confidence interval.

^d^Body mass index [kg/m^2^].

^e^Fasting plasma glucose.

**Table 3:** Risk factors for NODAT^a^ in patients receiving EPO^b^ ≤6,000 IU/week: multivariate analysis.

Variable	Univariate analysis			Multivariate analysis		
	OR^c^	95% CI^d^	*P* value	OR	95% CI	*P* value
Recipient age [y]	1.05	0.99–1.11	0.10	1.05	0.99–1.13	0.09
Recipient gender	2.59	0.61–17.8	0.21	3.91	0.75–33.8	0.11
Donor age [y]	0.96	0.91–1.03	0.24			
Pretransplant BMI^e^	0.98	0.76–1.24	0.84			
FPG^f^ [mg/dL]	1.02	0.94–1.09	0.68			
HbA1c [%]	8.32	1.74–47.2	<0.01	9.18	1.64–64.5	<0.05
Dialysis period [mo]	1.01	0.99–1.02	0.33			

^a^New-onset diabetes after transplantation.

^b^Erythropoietin.

^c^Odds ratio.

^d^95% confidence interval.

^e^Body mass index [kg/m^2^].

^f^Fasting plasma glucose.

**Table 4:** Sensitivity, specificity, positive predictive value, negative predictive value, and likelihood ratio of HbA1c for several cut-off values.

HbA1c	4.8	5.0	5.2	5.4	5.6
Sensitivity	91	73	64	45	27
Specificity	36	57	78	85	92
Positive predictive value	18	20	30	31	33
Negative predictive value	96	93	94	91	89
Likelihood ratio	0.21	0.25	0.44	0.45	0.5
